# Brain endothelial cells as phagocytes: mechanisms and implications

**DOI:** 10.1186/s12987-025-00637-w

**Published:** 2025-04-01

**Authors:** Rudy T. Chang, Mark J. Fisher, Rachita K. Sumbria

**Affiliations:** 1 Department of Biomedical and Pharmaceutical Sciences, School of Pharmacy, Chapman University, Irvine, CA USA; 2 Department of Neurology, University of California, Irvine, Irvine, CA USA; 3 Departments of Anatomy & Neurobiology and Pathology & Laboratory Medicine, University of California, Irvine, Irvine, CA USA

**Keywords:** Brain endothelial cells, Blood-brain barrier, Phagocytosis, Phosphatidylserine, Erythrophagocytosis, Erythrocyte, Red blood cell, Angiophagy

## Abstract

Brain microvascular endothelial cells (BECs) lining the brain capillaries form the anatomical site of the blood-brain barrier (BBB), providing a highly selective barrier to support brain homeostasis and function. While the BBB acts as a barrier to immune cells and pathogens under normal conditions, BECs can facilitate their entry into the CNS via a phagocytosis-like mechanism. A similar process is now increasingly reported for a diverse set of cargos, resulting in the categorization of BECs as “non-professional” phagocytes and redefining the conventional view that these cells are functionally non-phagocytic. This review aims to summarize research demonstrating the capacity of BECs to phagocytose various cargos, including aged red blood cells (RBC), myelin debris, and embolic particles. Mechanistically, BEC phagocytosis can be triggered by the exposure of phosphatidylserine on RBC, expression of adhesion molecules such as ICAM-1 and VCAM-1 on BECs, cargo-opsonization, and/or involve BEC cytoskeleton remodeling. Phagocytic activity by BECs has significant clinical implications ranging from regulation of cerebral microvascular patency (particularly by contributing to and resolving capillary stalling), clearance of brain parenchymal debris, and brain parenchymal invasion by pathogens. Further, BEC phagocytosis of RBC, which represents a cell (RBC)-in-cell (BEC) phenomenon, is implicated in hemorrhagic lesions including cerebral microhemorrhages. This review aims to shed light on BEC phagocytosis as an important function within the brain microvascular system and will delve into the underlying mechanisms, discuss the clinical implications, and identify gaps in our understanding of this phenomenon.

## Background

The microvascular system of the brain is a deeply intricate network that delivers nutrients, removes unwanted products, and provides a barrier. Brain microvascular endothelial cells (BECs) lining the brain capillaries form and maintain the blood-brain barrier (BBB); the latter is a highly selective semi-permeable barrier that regulates the movement of molecules, ions, and cells between the blood and the brain while protecting the brain from toxins and pathogens [1, 2]. The unique barrier characteristics of the brain microvascular endothelium compared to the periphery are attributed to (a) restricted paracellular transport across the BBB owing to the presence of tight junctions, which consist of proteins such as claudins, occludin, and adhesion molecules; (b) low pinocytic activity and fenestrations; (c) presence of active transport mechanisms to facilitate the transport of molecules, which include glucose transporters such as GLUT1 (which supplies the brain with glucose), and efflux transporters such as P-glycoprotein (which help remove foreign substances and toxins from the brain) [3, 4]​; and (d) high enzymatic activity which regulates the metabolism of xenobiotics and toxins [5].

While the BBB acts as a barrier to immune cells and pathogens under normal conditions, BECs can also facilitate their entry into the central nervous system (CNS), a key step in the development of CNS inflammation and infections [6–8]. Immune cell invasion into the CNS is extensively studied and occurs via a well-orchestrated multistep process involving contact, activation of endothelial cells, immune cell arrestation and migration, and transmigration to recruit peripheral immune cells to the CNS [7]. Modulation of immune cell trafficking is critical in neuroprotective responses and the pathogenesis of various neuroinflammatory disorders [7]​​. Similarly, pathogens, including bacteria, can enter the CNS through the paracellular or transcellular routes or by transmigration of a pathogen-infected immune cell, resulting in CNS infections [6, 8]. Among these mechanisms, the transcellular passage of bacteria is the most widely reported and involves the interaction of the BEC with the pathogen, pathogen engulfment via a phagocytosis-like endocytic mechanism involving actin cytoskeleton rearrangement, and subsequent migration across the BEC into the brain parenchymal space [6, 8].

The properties of immune cell and pathogen recognition and engulfment by the BECs for CNS entry are well-known and previously reviewed [6–8]. In addition, there is a growing body of literature showing that BECs exhibit a phagocytic phenotype for diverse micron-sized cargos, a trait traditionally associated with immune cells. In this review, we summarize these studies showing evidence of brain endothelial phagocytosis for diverse cargos (excluding pathogens, which have already been reviewed [6, 8]), underlying mechanisms, clinical implications of this phenomenon, and the gaps in our knowledge.

## Brain endothelium and phagocytosis– evidence from rodent and *in vitro* models and human studies

Macrophages and endothelial cells share several functional similarities, particularly in their roles in immune surveillance and tissue homeostasis. Both cell types exhibit phagocytic activity, though to different extents, allowing them to clear pathogens, apoptotic cells, and debris from tissues [9, 10]. Macrophages, classified as professional phagocytes, are specialized cells whose primary function is to engulf dead cells, pathogens, and debris [10]. Although phagocytosis by BECs is not traditionally associated with brain microvascular function, research findings indicate that endothelial cells, sometimes referred to as ‘non-professional phagocytes’, frequently perform phagocytosis to maintain tissue homeostasis, clear apoptotic cells, or act as part of immune defense, much like professional phagocytes albeit less efficiently [9]. Both macrophages and endothelial cells express pattern recognition receptors (PRRs), enabling them to detect and respond to pathogen-associated molecular patterns (PAMPs) and damage-associated molecular patterns (DAMPs) [10, 11]. This capability allows them to initiate inflammatory responses by producing cytokines and chemokines that recruit immune cells to sites of infection or injury [10, 11]. Moreover, both cell types contribute to angiogenesis and tissue remodeling, with macrophages playing a role in secreting pro-angiogenic factors and endothelial cells forming new blood vessels necessary for tissue repair [12, 13]. Interestingly, the functional similarities of professional phagocytes, such as macrophages, and ‘non-professional’ phagocytes, such as endothelial cells, are less surprising when considering that they are derived from erythromyeloid progenitor cells [14]. These progenitor cells are known to differentiate into macrophages/microglia but also serve as an additional source for endothelial cells forming the vasculature in different organs, including the brain, liver, and lungs [14, 15].

### BEC phagocytosis in rodent models and cell culture systems

**Fig. 1:**
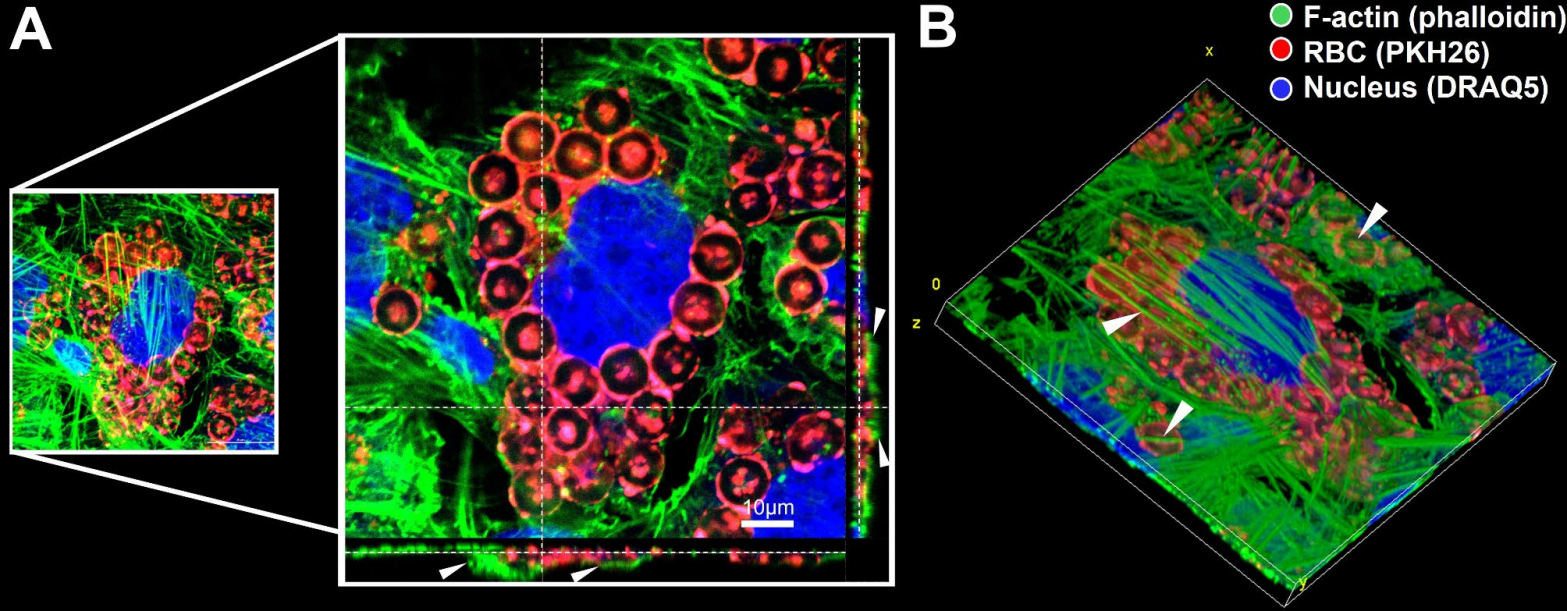
Human brain microvascular endothelial cells (hCMECD3 cells) were incubated with PKH-26-labeled human RBC (red) treated with 3mM tert-butyl hydroperoxide (t-BHP), an oxidative stressor. After 48 h, F-actin was stained using phalloidin (green) and nucleus was stained using DRAQ5 (blue), cells were fixed, and coverslips were imaged using confocal microscopy. The image shows robust uptake of tBHP-RBC by hCMECD3 cells. White arrowheads in the orthogonal view (**A**) and 3D reconstruction of the z-stack images (**B**) show actin covering RBC confirming RBC engulfment. Scale bar = 10µm.

Nearly four decades ago, studies highlighted the phagocytic ability of peripheral endothelial cells in the liver [16]. These liver endothelial cells lining the liver sinusoids were found to phagocytose various particulate ligands, including micron-sized latex particles [16], apoptotic bodies [17], and gold particles [18]. In addition to the fenestrated liver endothelial cells, Fens et al. reported evidence of phagocytosis by other endothelial cells. Their series of studies involved oxidatively-stressed red blood cells (RBC) with externalized phosphatidylserine (PS) and reduced deformability, both characteristics of aged RBC [19–21]. Peripheral endothelial cells were engaged in erythrophagocytosis of aged RBC under both static and flow conditions in vitro using human umbilical vein endothelial cells, and in tumor-bearing mice in vivo [19–21]. Accordingly, murine (bEnd.3 cell line) [22, 23], human (hCMECD3 cell line, Fig. 1A), and induced pluripotent stem cell-derived human [24] BECs retained a similar phagocytic phenotype for oxidatively stressed, PS-exposing RBC in vitro. BECs were engaged in significant tethering and uptake of PS-exposing aged RBC which increased intracellular and abluminal RBC-derived iron and hemoglobin without disrupting BEC monolayer integrity [22, 23]. A follow-up in vivo study confirmed increased RBC-BEC interaction following intravenous injection of PS-exposing aged RBC in mice. Increased RBC-BEC interactions in vivo were associated with RBC engulfment and migration across brain capillaries, a robust microglial response, and significant microglial association with the brain capillaries [25].

BEC-mediated erythrophagocytosis has now been observed for glycated and aged RBC, Plasmodium falciparum-infected RBC, and RBC exposed to zinc nanoparticles [26–28] using human BECs in vitro. Human RBC glycated and aged in vitro by incubating with different concentrations of D-glucose for 5 days showed increased internalization within human (hCMECD3) BECs [26]. Moreover, incubation of zinc-oxide nanoparticle-treated human RBC with human (hCMECD3) BECs increased intracellular hemoglobin confirming erythrophagocytosis by human BECs [27]. Furthermore, Plasmodium falciparum-infected RBC were internalized by human BECs, which was demonstrated using both the hCMECD3 cell line and primary human BECs [28]. Migration of infected RBC across the BECs was further confirmed using an in vitro human 3D spheroid model [28].

Phagocytosis targets of BECs extend beyond RBC. A seminal study by Lam and colleagues introduced the concept of angiophagy, a two-step process where brain capillary endothelium first engulfs emboli, followed by their transmigration into the brain [29]. After intracarotid injection into mice, fluorescently-labeled fibrin clots were partially cleared by hemodynamic forces or the fibrinolytic system in the early hours post-occlusion. However, the efficiency of clot washout decreased over time as microemboli were predominantly engulfed by brain capillaries, likely preventing their washout. Approximately 24 h after occlusion, the engulfed fibrin clots translocated across the brain capillaries, leading to recanalization of the occluded vessel [29]. In addition to fibrin clots, studies have reported that BECs can engulf other microemboli not susceptible to fibrinolysis, such as cholesterol emboli and polystyrene microspheres, facilitating their egress into the brain via angiophagy in mice and rats [30–32].

**Fig. 2:**
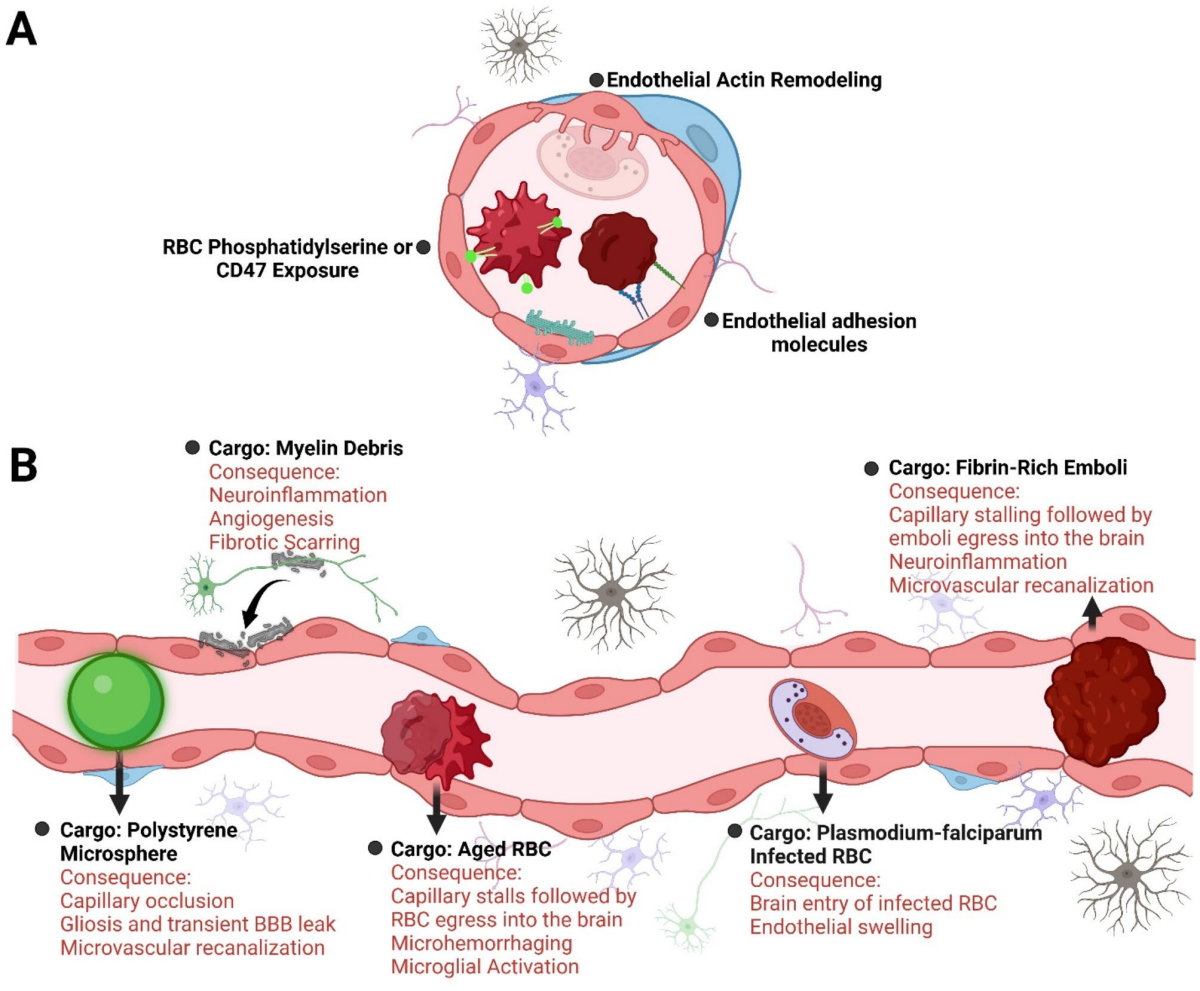
Reported mechanisms of brain endothelial cell phagocytosis range from recognition of phosphatidylserine exposure and CD47 on aged red blood cell surface, to detection by specific adhesion molecules such as intercellular adhesion molecule 1 (ICAM-1) and vascular cell adhesion molecule 1 (VCAM-1) that facilitate binding of infected red blood cell, and actin remodeling to form extensions that engulf emboli (**A**). Brain endothelial cells can perform phagocytic functions to maintain vascular patency or debris/cargo clearance (**B**). Brain microvascular endothelial cells can engulf exogenous particles including polystyrene microspheres, and clear aged/damaged or infected red blood cells, remove myelin debris, and maintain microvascular patency by phagocytosing emboli. While the primary consequence of brain endothelial phagocytosis appears to be cargo clearance, this process is also associated with neuroinflammation, transient hypoxia and BBB changes, and/or cerebral microhemorrhages.

Besides their role in performing phagocytic functions on the luminal side of the brain microvasculature, BECs also perform engulfment and phagocytosis of debris present on the abluminal side (within the brain parenchyma). An elegant study by Zhou and colleagues highlighted the phagocytosis and degradation of myelin debris by mouse BECs (primary cells and bEnd.3 cell line) primarily through the autophagy-lysosomal pathway in vitro. Myelin debris containing microvessels were also observed in a spinal cord injury (SCI) and experimental autoimmune encephalomyelitis (EAE) mouse model, confirming myelin debris phagocytosis by microvessels after demyelination in vivo [33]. Follow-up studies have consistently showed myelin debris phagocytosis by bEnd.3 cells, in vitro [34–36], and in a mouse model of white matter injury, in vivo [35]. These studies demonstrate that besides glial cells and macrophages, BECs also play a significant role in myelin debris phagocytosis. Overall, phagocytic activities by BECs have been increasingly reported for different cargo, leading to BECs being classified as ‘non-professional’ phagocytes [37]. This phenomenon appears to function to engulf and clear emboli, pathogens, cellular debris, and even intact RBC, both on the luminal and abluminal brain surface, providing an additional layer of protection to the CNS ​ (Fig. 2).

### BEC phagocytosis in human tissues

As discussed above, though phagocytosis by BECs has been largely demonstrated in vivo in rodents and using immortalized and primary human BECs in vitro, studies have shown the occurrence of this phenomenon in human tissues [26–28]. Brain sections prepared from postmortem brain tissues derived from two fatal cerebral malaria cases showed swelling and deformation of BEC using May-Grünwald-Giemsa stain. Further, Plasmodium falciparum-infected RBCs were found internalized by BECs in both cases of cerebral malaria [28]. Similarly, Grutzendler et al., demonstrated angiophagy in humans [31] as follows: Consecutive images from patients with retinal artery occlusion showed microvascular embolization by cholesterol crystals. Subsequent imaging at 3, 32, and 52 weeks after the initial imaging revealed migration of the emboli into the perivascular space adjacent to the occluded vessel. Vessel recanalization was confirmed using fluorescein angiography. This study suggests the occurrence of angiophagy in humans, and further studies confirming BEC-phagocytosis in human tissues are needed.

## Mechanisms of phagocytosis by brain endothelial cells

### Mechanisms involved in phagocytosis of RBC and emboli

The mechanistic machinery used by the BECs for phagocytosis shares commonalities with that used by peripheral endothelial cells and ‘professional phagocytes’ [38]. In this regard, loss of RBC lipid membrane symmetry (e.g., externalization of PS on the outer membrane of the RBC surface), reduced deformability, and oxidative stress are known to trigger erythrophagocytosis [39, 40]. Similarly, PS exposure and increased oxidative stress increased attachment and engulfment of aged and glycated RBC by BECs in vitro [22, 23, 26]. The treatment of RBC with tert-butyl hydroperoxide (t-BHP) or increasing D-glucose concentrations increased the formation of reactive oxygen species (ROS) and PS exposure, which significantly enhanced RBC adhesion and engulfment by mouse (bEnd.3) [22] or human (hCMECD3) [26] BECs, respectively. Similarly, zinc-oxide nanoparticle-treated and polystyrene nanoplastic-treated RBC underwent increased erythrophagocytosis via a PS-dependent mechanism [27, 41]. Consistent with these findings, in vivo injection of fluorescently-labeled PS-exposing t-BHP-treated RBC showed increased attachment to the brain capillaries with RBC egress into the brain parenchyma in mice [25]. Moreover, masking exposed PS with Annexin V or ethylene glycol tetraacetic acid and treatment with Vitamin C, a ROS scavenger, significantly reduced the engulfment of t-BHP-treated RBC by mouse BECs [23] or zinc-oxide nanoparticle-treated RBC by human BECs [27], respectively. These studies demonstrate the significant role of increased oxidative stress and RBC-PS exposure in initiating erythrophagocytosis by the BECs.

PS-mediated erythrophagocytosis by macrophages and peripheral endothelial cells is mediated by scavenger receptors (including stabilin-1 and stabilin-2), CD36, or PS receptors, and their role in BEC-mediated erythrophagocytosis needs to be determined [38, 42]. Notably, complete reversal of PS exposure was not sufficient to fully abolish erythrophagocytosis by BECs [23], indicative of additional mechanisms underlying phagocytosis of RBC by BECs. In this regard, cargo rigidity and geometry are critical factors that regulate phagocytosis by macrophages [43] and peripheral endothelial cells [19]. Oxidative stressors, t-BHP, and glycation reduce RBC deformability and increase erythrophagocytosis by peripheral endothelial cells [22, 26, 44]. Therefore, reduced RBC deformability may be an important factor regulating BEC-mediated erythrophagocytosis.

Besides PS, CD47 on RBC surface is a well-studied ‘do not eat me’ signal that blocks erythrophagocytosis by interacting with signal-regulatory protein alpha (SIRPα) on the surface of phagocytes [45, 46]. A reduction in RBC CD47 is suggested to increase peripheral RBC clearance [47] and is implicated in faster hematoma resolution by microglia/macrophages after intracerebral hemorrhage [48, 49]. Accordingly, t-BHP-treated RBC undergoing robust brain endothelial erythrophagocytosis showed reduced CD47 expression [23], supporting the role of RBC-CD47 in brain endothelial erythrophagocytosis.

Studies also show a pro-phagocytic role of RBC CD47. With RBC aging, RBC CD47 ‘do not eat me’ signal undergoes a conformational change and switches to a pro-phagocytic ‘eat me’ signal enhancing aged RBC clearance [45, 50]. A similar switch of CD47 to a ‘pro-phagocytic’ signal to facilitate phagocytosis of apoptotic cells is also reported [51]. Accordingly, CD47 showed a conformational change in aged and glycated RBC undergoing erythrophagocytosis by human BECs, suggesting that CD47 may also serve as an ‘eat me’ pro-phagocytic signal mediating brain endothelial erythrophagocytosis [26].

The role of adhesion molecules in the docking and transmigration of leukocytes into the brain parenchyma is well-known [52]. Similarly, intercellular adhesion molecule 1 (ICAM-1) and vascular cell adhesion molecule 1 (VCAM-1) are involved in increased adhesion of RBC to the vasculature under pathological conditions [38]. Consistent with these observations, and specific for the brain microvasculature, ICAM-1 was shown to serve as a docking site for Plasmodium falciparum–infected RBC, driving their internalization and transmigration into the brain tissue, albeit less efficiently than for leukocytes [28]. Adhesion molecules (including ICAM-1, PECAM-1) also increased the cytoadherence of Plasmodium knowlesi-infected RBC to human BECs, in vitro [53]. These observations suggest that proinflammatory conditions known to increase the expression of adhesion molecules on the BECs may potentiate erythrophagocytosis [54]. Accordingly, TNFα stimulation resulted in a dose-dependent increase in brain endothelial erythrophagocytosis in vitro [23].

Though the precise molecular mechanisms and specific receptors involved in brain endothelial erythrophagocytosis are still being studied, based on the above evidence and our understanding of this process by professional phagocytes, the following can be suggested: First, there must be recognition by the BECs of the RBC for binding to occur. PS-mediated recognition of the RBC by the BECs can be mediated by scavenger receptors [38]. Besides PS, adhesion molecules, including ICAM-1 and VCAM-1, may participate in cell-cell interaction that may be involved in the recognition and adhesion of RBC [38]. This initial tethering to the BECs is followed by other mechanisms that trigger phagocytosis, for example, via CD47 and/or RBC engulfment by the BEC; the latter is facilitated by the formation of pseudopodia-like protrusions that surround and enclose the RBC [55]. Phagosome formation occurs when the RBC are encapsulated and can fuse with lysosomes for breakdown of the RBC [56]. The mechanisms underlying the egress of intact RBC across the BEC are unknown but may be similar to the transmigration of leukocytes or pathogens [8], largely involving the BEC cytoskeleton, including actin remodeling. These mechanisms are involved in the engulfment and egress of diverse emboli by the BEC, with matrix metalloproteinases serving as important mediators of this process [29, 31, 32, 57].

### Mechanisms involved in phagocytosis of myelin debris

With respect to myelin debris phagocytosis, mouse BEC-derived microvessels from primary cells, and bEnd.3 cell line showed a time-dependent increase in engulfment of fluorescently-labeled myelin debris in vitro [33]. Myelin debris was attached to or engulfed by the microvessels or translocated to the luminal membrane. Key receptors, complement-3 receptor (CR3), Mac-2 (Galectin-3), and low-density lipoprotein receptor–related protein 1 (LRP1), involved in myelin debris engulfment by macrophages/microglia, were not involved in BEC-mediated myelin debris engulfment [33]. Instead, myelin-debris opsonization was key to BEC-mediated myelin debris engulfment. BECs were shown to internalize and degrade myelin via an Fc-receptor-based mechanism and only antibody-opsonized myelin debris but not “naked” myelin debris was marked for phagocytosis by BECs [33, 34]. Once phagocytosed, myelin debris was degraded within the BECs by the autophagy-lysosomal pathway instead of the endocytosis-lysosomal pathway, with histone deacetylase 6 (HDAC6) regulating this process [33, 34]. RNA-sequencing showed differentially expressed genes largely relevant to extracellular matrix formation, vesicle trafficking, inflammation, and endothelial angiogenesis and permeability in myelin debris-positive BEC, compared with naïve BECs [33].

## Clinical implications of brain endothelial phagocytosis

### Role in capillary stalling and perfusion

Brain endothelial erythrophagocytosis, a phenomenon by which aged/damaged RBC attach to and are engulfed by the brain capillary endothelium, may be a fundamental mechanism involved in the increased interactions and clearance of RBC at the brain capillary level [25]. The initial stages of brain endothelial erythrophagocytosis involve increased RBC interactions with the brain endothelium, which is relevant to brain capillary stalling, followed by brain endothelial erythrophagocytosis-mediated RBC clearance; the latter has relevance in the resolution of capillary stalling mediated by RBC to maintain cerebral capillary patency. Increased RBC-BEC interactions may lead to brain capillary stalls [38, 58, 59] given that the diameter of human or mouse RBC is approximately 6–8 μm, while the brain capillary diameter may be as small as 2 μm [60–62]. Therefore, under normal conditions, RBC transit through brain capillaries is achieved by their ability to deform, which is altered with disease and physiological aging and is likely to increase susceptibility to brain capillary stalling [63]. Additionally, increased RBC adherence to the brain capillary endothelium due to pathology and/or aging can increase brain capillary stalling [38]. Accordingly, intravenous injection of t-BHP-treated aged RBC in mice significantly increased capillary stalling and reduced cerebral blood flow velocity, followed by spontaneous resolution of capillary stalling and normalization of cerebral blood flow velocity with RBC egress into the brain parenchyma [25].

The role of brain endothelial erythrophagocytosis in capillary stalling and maintenance of capillary patency has significant clinical implications as interruptions to microvascular blood flow are associated with normal aging and pathological conditions, including stroke, Alzheimer’s disease (AD), and cognitive impairment [64, 65]. In stroke neurology, cerebral capillary stalling is often associated with the ‘no-reflow’ phenomenon wherein continued penumbral microvascular disturbances and tissue damage are observed despite macrovascular patency [66–68]. Experimental evidence from stroke models shows that brain capillary stalls, mediated largely by neutrophils but also by RBC [69–71], are associated with the no-reflow phenomenon and increased brain damage and functional deficits [66, 68]. In AD, brain capillary plugging by RBC has been documented in mice [72, 73]. Further, RBC damage/alterations and increased RBC interactions with the brain capillary endothelium have been reported in AD [74–76]. These findings suggest that increased interactions between RBC and brain capillaries in AD can potentiate capillary stalling, reduce cerebral blood flow, and may increase AD pathology and cognitive decline [77].

Similar to brain endothelial erythrophagocytosis, angiophagy is implicated in brain microvascular occlusion and recanalization following embolic microvascular occlusion [29, 31, 57]. Following intracarotid injection, microemboli lodged in the brain microvasculature were engulfed by BECs, limiting their access to fibrinolysis and preventing early washout. This was followed by emboli translocation outside the microvasculature into the brain parenchymal space, leading to the recanalization of the stalled brain capillary and re-establishment of blood flow [29, 31, 57]. Brain microvascular embolization led to transient hypoxia near the capillary stall, which resolved with the reestablishment of blood flow following angiophagy [29, 78]. The extent of hypoxic injury due to brain capillary stalling depended on the size of the microemboli such that greater injury, and even infarction, was noted with larger (25–50 μm) microspheres [78]. Furthermore, angiophagy was significantly reduced in aged mice indicating possible age-related changes to the BEC-mediated phagocytosis [29].

These studies collectively support BEC phagocytosis of RBC or emboli as mechanisms that help maintain capillary patency, the latter being critical to brain health. A delay or failure in BEC phagocytosis of RBC or microemboli, either due to aging or pathology, may increase brain capillary stalls. The latter may be an important contributor to reduced microvascular blood flow, with potential consequences of brain hypoperfusion and ischemic injury.

### Role in cerebral hemorrhagic and parenchymal injury

Though studies largely support the protective effects of brain endothelial phagocytosis of either RBC or diverse emboli via maintenance of capillary patency, changes to the brain microvasculature and within the brain parenchyma have been reported. For example, during brain endothelial erythrophagocytosis, once engulfed, the iron-rich RBC or their degradation products migrate into the brain parenchyma, trigger neuroinflammation, and are taken up by microglia, forming the pathological substrates of cerebral microbleeds [23, 25]. Further, brain endothelial erythrophagocytosis is reported to increase intracellular iron and hemoglobin within the BEC [23, 27], which may disrupt BEC function, further aggravating hemorrhagic injury. Brain endothelial erythrophagocytosis may, therefore, result in both ischemic (discussed in the section above) and hemorrhagic consequences. With respect to emboli extravasation into the brain parenchyma, angiophagy produced transient endothelial dysfunction to allow for emboli egress into the brain parenchyma. This process was also associated with a temporary and focal loss of dendritic spines, mild gliosis, and hypoxia (discussed in the section above) [29, 32, 78], changes that were more pronounced with aging [29]. Collectively, these studies indicate that brain endothelial phagocytosis can lead to parenchymal injury beyond the microvasculature.

### Role in demyelinating disorders and cerebral malaria

BECs have been reported to engulf and degrade myelin debris, likely supplementing clearance by professional phagocytes such as macrophages and microglial cells [33–35]. While myelin debris clearance is crucial for remyelination and resolution of inflammation, brain endothelial engulfment and clearance of myelin debris promote neuroinflammation, abnormal angiogenesis, and fibrotic scar formation, leading to secondary injury in SCI and EAE mouse models of demyelinating disorders [33]. In a mouse model of hypoperfusion-induced white matter injury, myelin phagocytosis by the BECs was associated with iron overload, cell death due to ferroptosis, and subsequent BBB disruption. This inhibited iron transport across the BBB impacting iron delivery to the brain and hindering white matter repair [35]. Interestingly, the engulfment of myelin debris by BECs in vitro promoted endothelial-to-mesenchymal transition, reduced adhesion between adjacent BECs and increased BEC migration [36].

From the perspective of cerebral malaria, brain endothelial erythrophagocytosis facilitated parasite ingress into the brain by enabling the entry of Plasmodium falciparum-infected RBC. This contributed to the pathology of cerebral malaria [28]. The mechanism of parasite entry into the brain appears similar to the Trojan horse route reported for bacterial invasion of the CNS, wherein bacteria-infected leukocytes carry the pathogen into the brain [6].

## Brain endothelial erythrophagocytosis: A cell-in-cell phenomenon?

Studies of brain endothelial erythrophagocytosis show intact t-BHP-treated (biochemically aged) RBC within BECs, and several RBC within one BEC [22]. This phenomenon appears to be similar to the cell-in-cell phenomenon first described in the early to mid-1900s [79]. Cell-in-cell is a process of cell entry (effector cell) into another cell (target cell) resulting in diverse fates including the destruction of the effector cell by the target cell, destruction of the target cell, or escape or release of the effector cell by the target cell [79].

Different forms of cell-in-cell phenomenon have been reported. For example, lymphocytes are found to co-inhabit other living cells by ‘emperipolesis’, a widely observed phenomenon by cancer cells [80]. Besides emperipolesis, which is a heterogenous cell-in-cell phenomenon, entosis is a homogenous cell-in-cell phenomenon [81]. While emperipolesis and entosis result in effector cell clearance, effector cells may escape the target cell via transcytosis. Therefore, applying these principles to studies on brain endothelial erythrophagocytosis, uptake of aged RBC by the BECs may represent a cell-in-cell phenomenon with different fates: degradation of the RBC within the BECs (cell-in-cell death) to clear RBC stalls, loss of the BEC due to increase in intracellular iron resulting in microvascular disruption, and/or escape of RBC from the BECs into the brain parenchymal space leading to hemorrhagic lesions. These fates may occur simultaneously and are not necessarily mutually exclusive.

## Conclusions

BEC-mediated phagocytosis is increasingly reported on the luminal and abluminal surfaces of the brain capillaries. On the capillary luminal surface, BECs phagocytose diverse cargo, ranging from fibrin-rich emboli and microspheres to altered RBC. On the abluminal surface, BECs can phagocytose myelin debris. Phagocytosis by BECs may have a substantial impact on maintaining microvascular patency and serve as an important entry and/or clearance mechanism in the brain. Alterations in this process with aging or pathology can potentially directly impact brain microvascular ischemic or hemorrhagic injury.

Though this review highlights the investigations on the mechanisms and implications of brain endothelial phagocytosis, fundamental questions remain. First, since brain endothelial phagocytosis is reported for such diverse cargos, it is important to elucidate the commonalities and differences in mechanisms that trigger phagocytosis of these cargos. The role of cargo size, rigidity, and mechanosensing in triggering phagocytosis by brain endothelium may be relevant in this regard. Second, the impact of physiological aging on BEC-mediated phagocytosis, along with the fate of the ingested cargo and BEC after phagocytosis, are poorly defined. Third, the contribution of BEC-mediated phagocytosis as a mechanism supplementary to microglial clearance within the brain parenchyma needs further investigation. Finally, further studies investigating BEC-mediated phagocytosis in human tissues are needed to delineate the physiological and clinical implications of this phenomenon. Addressing these knowledge gaps is likely to reveal novel mechanisms and crucial therapeutic targets, providing new insights into our understanding of the pathogenesis of cerebral microvascular and parenchymal injury.

## Data Availability

All data generated or analyzed during this study is available within the original papers or on reasonable request.

## Abbreviations

AD: Alzheimer’s disease
BBB: Blood-brain barrier
BEC: Brain microvascular endothelial cell
CNS: Central nervous system
DAMPs: Damage-associated molecular patterns
GLUT-1: Glucose transporter 1
HDAC6: Histone deacetylase 6
ICAM-1: Intercellular adhesion molecule 1
PAMPs: Pathogen-associated molecular patterns
PRRs: Pattern recognition receptors
PS: Phosphatidylserine
RBC: Red blood cells
ROS: Reactive oxygen species
SIRPα: Signal-regulatory protein alpha
t-BHP: Tert-butyl hydroperoxide
VCAM-1: Vascular cell adhesion molecule 1
